# The future of the Affective Neuroscience Personality Scales: A reflection on seven pressing matters

**DOI:** 10.1017/pen.2022.2

**Published:** 2022-09-20

**Authors:** Christian Montag, Mark Solms, Christine Stelzel, Kenneth L. Davis

**Affiliations:** 1 Department of Molecular Psychology, Institute of Psychology and Education, Ulm University, Ulm, Germany; 2 Neuroscience Institute and Psychology Department, University of Cape Town, Rondebosch, Western Cape 7701, South Africa; 3 General Psychology and Neurocognitive Psychology, International Psychoanalytic University Berlin, Berlin, Germany; 4 Pegasus International, Greensboro, NC, 27408, USA

**Keywords:** ANPS, Panksepp, Trait-state debate

## Abstract

The Affective Neuroscience Personality Scales (ANPS) were designed to provide researchers in the mental sciences with an inventory to assess primary emotional systems according to Pankseppian Affective Neuroscience Theory (ANT). The original ANPS, providing researchers with such a tool, was published in 2003. In the present brief communication, about 20 years later, we reflect upon some pressing matters regarding the further development of the ANPS. We touch upon problems related to disentangling traits and states of the primary emotional systems with the currently available versions of the ANPS and upon its psychometric properties and its length. We reflect also on problems such as the large overlap between the SADNESS and FEAR dimensions, the disentangling of PANIC and GRIEF in the context of SADNESS, and the absence of a LUST scale. Lastly, we want to encourage scientists with the present brief communication to engage in further biological validation of the ANPS.

*The Affective Neuroscience Personality Scales* (ANPS) were published in 2003 (Davis et al., 2003). Jaak Panksepp wanted to provide human personality researchers and clinicians with a free self-report assessment tool measuring the influence of six of the seven documented primary emotions (SEEKING, ANGER, FEAR, CARE, SADNESS, and PLAY) (Davis & Montag, 2019; Panksepp, 1998). In that initial report, the Big Five scales (Extraversion, Agreeableness, Conscientiousness, Emotional Stability, and Openness) were the validation focus for the ANPS. In 2011, Davis and Panksepp published a second article, which reviewed intervening publications concerning the ANPS and formally introduced the new “ANPS 2.4,” with improved psychometric properties (Davis & Panksepp, 2011). The ANPS 2.4 included 11 completely new items and 14 reworded items for the 6 primary emotion scales and 1 new item for the Spirituality scale. In addition, 10 of the original filler items were replaced with seven Social Dominance and three Social Anxiety items. The addition of a short Social Dominance scale was in recognition of the important role of Social Dominance in human personality and in social organizations – as well as its biological roots – a topic that was later thoroughly addressed by van der Westhuizen and Solms (2015). A further revised edition of the ANPS (Montag et al., 2021) shifted to a six-point response scale in order to increase internal consistencies, and it replaced seven items: One Social Dominance item, one experimental FEAR item, and five experimental SADNESS items. The revision focused on further increasing the scale Cronbach’s alpha reliabilities, augmenting the Social Dominance scale, and attempting to further decrease the correlation between the FEAR and SADNESS scales.

By this time, the ANPS had been translated into two dozen languages, many of which had been validated against the Big Five/Five Factor Model. Davide Marengo and colleagues worked through this material and identified 21 studies in 10 different languages with 10 000 subjects suitable for a meta-analysis (Marengo et al., 2021). Remarkably, this work confirmed the large correlations in the original 2003 paper which was based on 171 students: Extraversion correlated with PLAY, Agreeableness with high CARE and inversely with ANGER, Openness to Experience with SEEKING, and Emotional Stability inversely with FEAR, SADNESS, and ANGER. In addition, there were no high correlations with Big Five Conscientiousness, confirming that Conscientiousness was most likely not a primary emotion. The Marengo et al. meta-analysis provided strong evidence in support of Panksepp’s belief that primary emotions form the foundation of personality and psychopathology and serve as a template for expanding our interpretation of the Big Five. For more detail on the primary emotions, see the following works in the brackets (Montag & Davis, 2020; Panksepp, 2006; Panksepp & Biven, 2012). In the remainder of this paper, we want to reflect on key avenues to improve assessment of the primary emotional systems according to Pankseppian Affective Neuroscience Theory (ANT) to ensure progress in this highly relevant research field, spanning the interests of neuropsychoanalysts, personality psychologists, and experimental affective neuroscientists.

## 1. Traits and states

From its inception, the ANPS was designed to measure relatively stable, enduring emotional characteristics, in other words, traits. Many of the items ask about how one typically responds in various situations, such as “I am known as one who keeps work fun.” As discussed above, the ANPS has been validated against other trait assessments, whether seen as descriptive Big Five inventories (Goldberg, 1993) or as the Five Factor Model (McCrae & Costa, 1987). As reviewed in Montag et al. (2021) the ANPS scales have been used as trait measures to describe and confirm the emotional endophenotypes of psychopathologies, from bipolar spectrum disorders (Savitz et al., 2008a, b) to personality disorders (Karterud et al., 2016). Panksepp‘s primary emotional systems, as traits, have been used to explain why certain persons are more prone to develop neuropsychiatric disorders such as depression (Montag et al., 2017) and ADHD (Wernicke et al., 2019).

This said, we believe that clinically oriented psychologists as well as those studying human happiness would profit from *state* measures of the primary emotional systems, as this would facilitate investigations of mental *change*, for instance, in patients undergoing treatment. Further, Panksepp encouraged the development of state variables corresponding to the current trait variables, to evaluate environmental and neurochemical research challenges (Panksepp, 2006, p. 781). In addition, Schimmack (2003) proposed measuring the frequency, intensity, and duration of affective experiences. Such approaches to state assessment could have applications in therapeutic settings, but there are other areas where the ANPS can be applied that would profit from an inventory assessing states of the human mind against the background of ANT (for an overview see Montag et al., 2021). A recent measure of primary emotions (Montag & Davis, 2018; Rozgonjuk et al., 2021), which might be used for such research, is the adjective-based ANPS-Adjective Ratings(AR) (24 items). In a similar example, (Anderson & Phelps, 2002) reported 30 daily ratings of the 20 PANAS adjectives between a patient with bilateral amygdala damage and 2 matched controls and concluded that the amygdala was not necessary for the subjective experience of *negative or positive* affective states. However, it is clear that much research in this area still needs to be done to bridge trait and state measures in the context of Pankseppian ANT.

## 2. Factor analysis and length of the ANPS

A second area warranting more thought touches upon factor analytic issues and the length of the ANPS. First of all, although the ANPS has been validated in many settings (again see the recent review by Montag et al., 2021), its psychometric properties could be improved. If we take a look at the factor structure of the ANPS, it is robustly observed that the ANPS six primary emotion scales yield two factors called positive and negative emotions, which is somewhat troubling if it is true that there are six distinct primary emotional systems. However, note that six-factor ANPS solutions have been published, namely, Abella et al. (2011) with a Spanish version and Montag and Davis (2018) using personality descriptive adjectives.

This raises the question as to, how distinct these primary emotional systems really are. Although Panksepp demonstrated, with various experimental procedures including electrical brain stimulation, that six distinct primary emotions can be documented (for definition of a primary emotional system, see Panksepp, 2010), it is also true that some neuroanatomical and neurotransmitter/neuropeptide overlap exists between the systems, and the activity of the different systems have to be seen in concert with each other (see depression example above).Thus, one could ask, from a neuroscientist’s viewpoint, whether we actually expect six orthogonal systems to exist in a self-report questionnaire, which has been constructed on the basis of Panksepp’s theory. Please note that competing biological personality theories shedding light on basic emotions – such as Jeffrey Gray’s behavioral activation and inhibition systems (BAS/BIS), as measured by Carver and White’s questionnaire (1994) – are also robustly correlated with each other (negatively). This is also true for Cloninger’s temperament and character inventory (Farmer & Goldberg, 2008). This is a debate not to be solved easily, but perhaps investigations of the different personality theories in different life scenarios might provide an additional relevant layer of analysis.

One thing is less debatable: For many research purposes, the initial 14-item scales of the ANPS are too long. There is a need – in particular when working also with clinical groups – to have shorter sound measures of the ANPS. Some shortened versions have been developed previously, such as the short ANPS (Pingault et al., 2012) and the brief ANPS (Barrett et al., 2013), or the already mentioned adjective-based ANPS-AR (Montag & Davis, 2018). However, these measures need more validation studies to determine their suitability in different contexts including whether they adequately sample the affective spaces they attempt to measure.

## 3. Disentangling the “Protest” and “Despair” phases of separation distress

The SADNESS scale – as it currently exists in the ANPS – is not able to distinguish the different phases of separation distress. Watt and Panksepp (2009) argued that Bowlby’s description of a shift from “Protest” to “Despair” following social loss is the process whereby “patients transition from acute separation distress and sadness (short-lived depressive responses?) into chronic depression. Our working hypothesis is that brain regions that initially coordinate separation-distress and sadness reactions undergo some kind of neurodynamic shift over time to coordinate the shutdown of separation distress and the initiation of depression” (p. 28). Their hypothesis for such a mechanism was “underactive or disregulated SEEKING urges” (p. 40), which is exactly what (Montag et al., 2017) observed in their study looking at correlations between ANPS traits and Beck’s Depression Inventory (BDI) scores in both clinically depressed patients and healthy participants (a finding later supported by Fuchshuber et al., 2019). In both groups, those participants whose BDI score suggested they were more depressed tended to have both higher SADNESS scores and lower SEEKING scores, which was likely the trigger that shifted their SADNESS into a depressed disengagement with the world (or which suggested such a possibility for the healthy group). Basically, if the Protest phase does not result in reunion with loved ones, SEEKING activity is diminished while high SADNESS persists, resulting in possible depression. Especially for young people and animals, depression could be seen as an evolved energy-saving mode: The organism still hopes to be helped, but will not actively seek for help anymore, as this is too energy-consuming (and the crying might attract predators).

Aside from this, it is up for discussion whether it is meaningful and relevant to divide the SADNESS scale of the ANPS into Protest and Despair subscales (see above, PANIC vs. GRIEF). Such a division may take into account that humans potentially show stable individual differences in how prone they are to bouts of sadness that could cascade into depression, presumably independently from downregulation of SEEKING engagement. Of course, the Protest and Despair phases may also need to be described as SADNESS system states (the ANPS family of inventories mostly measures traits and not states), and in particular for clinically oriented psychologists it would be helpful to obtain measures which provide insight into whether a high Protest score combined with a high Despair score might point to possible depression, or whether a low Despair score would suggest the Protest phase might have run its course without developing into depression. However, the alternate model of depression discussed above (Watt & Panksepp, 2009) suggested that a dysregulated or underactive SEEKING system might be the mechanism that initiated depression rather than a shift into a Despair/Grief phase. This is an empirical issue that remains to be resolved.

## 4. FEAR versus SADNESS in the ANPS

A perceived problem of the ANPS is the consistently high correlation between the SADNESS and FEAR scales. One possible reason for this might be that the FEAR scale assesses persons who are prone to worry or experience anxiety, which also is inherent to SADNESS/PANIC, and excluding the more cognitive aspects of worrying from the SADNESS dimension could possibly help to lower correlations between the SADNESS and FEAR scales (e.g., “I tend to think about losing loved ones often”). Gray and McNaughton’s revised reinforcement sensitivity theory (McNaughton & Corr, 2004; Smillie et al., 2006) made the case that anxiety and fear are different emotions; whereas anxiety is a more cognitive emotion^1^, being triggered in uncertain situations, FEAR kicks in at times of unequivocal danger. To bring a classic example from the Blanchard and Blanchard lab illustrating the difference between anxiety and FEAR (e.g., Apfelbach et al., 2005): When a rat is put into a cage which has been prepared with cat odor, anxiety will be triggered (in terms of Gray the “BIS” is activated by conflicting information which *might* be fearful) alongside active monitoring of the situation. Again, the air smells of danger, but physically a cat is not there. This is a conflict for the mammalian brain. The rat will now carefully monitor the environment for more information to solve the conflict (is danger really near?). If the conflict is solved, either SEEKING activity is seen once more (clear exploration of the environment, Gray speaks here of the BAS) or FEAR results as an indication of a cat being actually there. Then – also depending on the defensive distance – the rat will show fight, flight, and/or freezing behavior. Considering the importance of the distinction between an anxious and a fearful state of mind, we believe it is important to include more items on FEAR (e.g., flight tendencies) in the ANPS (e.g., Reuter et al., 2015). This might help also to reduce the shared variance of SADNESS and FEAR, as is currently observed.

An alternative hypothesis is that the FEAR system and the SADNESS system are both very sensitive to pain. Although the FEAR system responds to *physical* pain and the SADNESS system to *psychological* pain, the two types of pain seem to be generated by similar brain circuits (MacDonald & Leary, 2005; Panksepp, 2003). Perhaps not remarkably, the physical and psychological pain systems appear to closely interact with each other: Increased social support (low SADNESS) has been shown to reduce experienced pain (low FEAR-related experience) and vice versa. For example, more social support results in less cancer pain (Zaza and Baine, 2002) and reduces labor pain (Kennell et al., 1991) and results in taking less pain medication following bypass surgery (Kulik & Mahler, 1989). Inversely, students who believe they performed poorly on an exam rated a cold pressor task as more painful (Hout et al., 2000; Levine et al., 1993). Further demonstrating the overlap between the phylogenetically older sensory pain system and the separation distress pain system, Eisenberger et al. (2006) used their virtual ball-tossing game to show that greater sensitivity to physical pain predicts the level of social rejection experienced, and conversely, the level of cyberball social rejection predicts greater sensitivity to heat-induced pain. So, it is possible that FEAR and SADNESS are evolutionarily linked by their shared pain experiences, which may render orthogonality of the scales difficult in general. A solution for this problem then would be difficult, as on brain neuroanatomical and biochemical levels an overlap exists in these systems, to some extent. Despite the evidence put forward regarding overlaps between the physical and psychological pain systems, we nevertheless want also to mention research contradicting this hypothesis by running fMRI pattern classifiers (Woo et al., 2014).

Further difficulties contributing to the links between FEAR and SADNESS are the symptoms shared by anxiety and depression disorders, such as “sleep disturbances, agitation, restlessness, irritability, difficulty concentrating, loss of control, fatigue, fear, distress and, of course, anxiety“ (Fuchs & Flügge, 2006, p. 324). Fuchs and Flügge go on to state that comorbidity between anxiety and mood disorders is the rule rather than the exception, citing Gorman (1996) to the effect that over 80% of adults with depression also have significant anxiety symptoms. Newer numbers stem for a recent published meta-analysis. Saha et al. (2021) report that “… there was substantial comorbidity between various mood and anxiety disorders with a median OR of 6.1 (range 1.5–18.7).” (p. 289). The bottom line is that it is small wonder that the ANPS FEAR and SADNESS scales correlate highly.

## 5. LUST

The original ANPS included no items for assessing individual differences in LUST. The decision not to include such items was based on the the “danger“ that at least some participants might answer in a socially desirable fashion and also that spillover effects on the remaining items might occur. This said, without assessing LUST in the ANPS, the Pankseppian primary process emotions are not assessed in a complete manner. There has been a LUST scale generated by Donné van der Westhuizen, centered around sexual desire and arousal, but it was never published. Walther van Lieshout started validating a Dutch translation of the ANPS 3.1 that would include a LUST scale, but that work is not yet completed. However, Fuchshuber et al. (2022, p. 8) just published a 12-item ANPS LUST scale with “item content clearly assess[ing] the individual capacity to attain sexual pleasure,” which may be a different focus than van der Westhuizen’s scale which centered on sexual desire and arousal. Fuchshuber et al. (2022, p. 8) reported that sexual desire “items were excluded based on initial item statistics and considerations regarding internal consistency,” but they left open the possibility of including items reflecting sexual urges in future versions. The authors of the present paper take the position that a LUST scale would be a useful addition to the ANPS – at least a useful option – but items should be formulated in a more general way to get insights into an active LUST system, and not asking about detailed sexual practices or sexual orientation. So, in general, we agree that for some projects, particularly in (neuro-)psychoanalysis, a LUST scale would be a necessary addition. However, the apparent content differences in the two existing LUST scales raise issues around what criteria are most suitable for selecting items and defining scale content for this scale, a topic discussed below, in the next section.

## 6. Validation of the ANPS against biology versus statistics – can we really measure our primary emotional systems via introspection?

Jaak Panksepp worked mainly with nonhuman species, and much of what he observed remains to be translated and tested in the context of the human brain. The recent review by Montag et al. (2021) shows that most studies using the ANPS do not include biological variables. We believe the strength of ANT is its grounding in brain science, providing scientists with falsifiable hypotheses on what brain areas, chemistries, and molecules underly individual differences in primary emotional states and traits. Applying high-field imaging techniques to measure brainstem-based primary emotional responses in humans and relating those to individual differences in ANPS self-report may be one avenue for future research. Also, the investigation of higher-order (cortical) activity in relation to specific ANPS scales or profiles may be of interest, and also connectivity patterns during rest (Deris et al., 2017) or the application of time-resolved electrophysiological methods to disentangle initial emotional responses from regulatory processes. Measuring facial actions and vocal prosody (Cohn et al., 2009) could also be added to the affective neuroscience tool chest. Beyond that, we might hint at new disciplines investigating digital footprints to gain insight into emotional life. This might be an additional interesting avenue for the future (Montag et al., 2022; Montag & Elhai, 2019).

On the statistical side, overreliance on the correlational analysis of batches of self-report instruments for evaluating ANPS scales clearly has limitations (in particular, when one wants to investigate biologically rooted primary emotional systems). Furthermore, Cattell’s faith in the capacity of factor analytic statistics to satisfactorily delineate personality source traits arising from the subcortical mammalian brain (namely, Panksepp’s primary process emotional action systems) must be questioned, especially when relying on verbal self-report responses generated by human tertiary cortical minds. This all said, the ANPS has been created on the basis of findings from Pankseppian ANT and brings the advantage of clearly testable neuroanatomical structures and brain chemicals, as presented among others in the paper by Montag and Davis (2018).

## 7. Format

Another question is whether alternative assessment formats should be explored. Up until now, most of the ANPS efforts have gone into the standard personality questionnaire style of generating hypothetical situationally written single statements intended to tap into a targeted emotion and asking participants to rate their personal level of endorsement of the item. However, that style has not always been the case, and the first demonstration of five reliable personality factors (Tupes & Christal, 1992) used 35 forced-choice trait measures derived by Raymond Cattell, using Gordon Allport adjectives with each pole of the trait consisting typically of 20 or more descriptive words. Further, most ratings in the Cattell era were provided by independent observers familiar with the subjects rather than by self-reports. But, for a more recent example, see Fleeson and Law (2015).

Pointing to an ANPS adjective-based assessment, Davis and Feren (see Montag et al., 2021) provided a conference poster illustrating the use of adjectives to assess Panksepp’s primary emotions. One result of this has been the ANPS-Adjective Ratings (Montag & Davis, 2018; Rozgonjuk et al., 2021). Such adjective rating scales can be useful for both the observer ratings and the comparison of self-ratings and observer ratings, yielding important insights. Adjective self-ratings can also be used for repeated ratings (perhaps daily) to measure frequency and intensity of subjective emotions, by using rating scales such as 0=not at all, 1=very slight intensity, and up to 6=extreme intensity (Diener et al., 1985; Schimmack, 2003). By aggregating repeated ratings, very high Cronbach’s alphas can be achieved. Combining self-ratings with observer rating (perhaps by the therapist) can validate the self-ratings or open up discussion when discrepancies occur. Repeated self-ratings within the context of therapy sessions could also document therapeutic progress.

## 8. Summary and solutions

The present work highlights several areas where the ANPS needs improvement. This does not mean that the ANPS as it stands now is without merit. Many existing studies as summarized in the recent work by Montag et al. (2021) show that the ANPS can be used both in a meaningful way in clinical settings, in personality science and in the neurosciences to shed light on a broad range of psychological phenotypes.

Which areas need to see improvement and what solutions might there be to the issues raised? In the present paper, we mention that state measures according to ANT are much needed and the ANPS-AR might be a first measure in this direction. Hence, testing the state version of the ANPS-AR might be a step toward establishing a state measure that also captures fluctuating imbalances of primary emotional systems in psychopathologies. Further empirical investigations of shorter and longer versions of the ANPS, including item reformulations, might help to further improve its psychometrics. This research must be carried out in both healthy and clinical samples, and particularly in depression research, where patients are often fatigued and might profit from a shorter version. Also from an economic perspective, shorter versions would be highly welcomed. Future research should try also to find solutions for the discussed issues surrounding the separation distress hypothesis and find a better disentanglement of the FEAR/SADNESS emotions. In our view, this can only be done by also formulating new items. For some scientists it might be a pressing issue to have a valid and reliable LUST scale, and we think that such a hopefully valid and reliable scale should give insights into individual differences in LUST activity. Of note, this does not imply asking about concrete sexual engagements with other persons, because one can have a highly active LUST system without any sex partner. The hardest part to tackle will most likely be the further biological validation of the ANPS scales, at best with different methods mapping the different layers of the mind, ranging from molecular genetics to functional magnetic resonance imaging (fMRI) and electroencephalography (EEG) studies. We ended this work with a discussion of item formats (sentences vs. adjectives; different answer formats), an area which also needs further attention and again touches upon psychometrics. We hope that the present work will encourage other scientists to see the potential of the ANPS and the ANT for their own research projects and join forces to improve the ANPS scales.

For more information on ANPS research, please visit www.anps-research.com.

## Footnotes

1 We stress the *more* in ‘more cognitive emotion’ and mean that while monitoring of the situation, the prefrontal cortex plays a pivotal role. This said, the study by Mobbs et al. (2007) demonstrated that while risk assessment is taking place, the lateral amygdala is also activated. Hence, anxiety as a cognitive emotion needs to be seen within the framework of the revised reinforcement sensitivity theory, as put forward in the text.
